# A Link between Meiotic Prophase Progression and Crossover Control

**DOI:** 10.1371/journal.pgen.0020012

**Published:** 2006-02-03

**Authors:** Peter M Carlton, Alfonso P Farruggio, Abby F Dernburg

**Affiliations:** 1 Life Sciences Division; Lawrence Berkeley National Laboratory; Berkeley, California, United States of America; 2 Department of Molecular and Cell Biology; University of California Berkeley, Berkeley, California, United States of America; Stowers Institute for Medical Research, United States of America

## Abstract

During meiosis, most organisms ensure that homologous chromosomes undergo at least one exchange of DNA, or crossover, to link chromosomes together and accomplish proper segregation. How each chromosome receives a minimum of one crossover is unknown. During early meiosis in Caenorhabditis elegans and many other species, chromosomes adopt a polarized organization within the nucleus, which normally disappears upon completion of homolog synapsis. Mutations that impair synapsis even between a single pair of chromosomes in C. elegans delay this nuclear reorganization. We quantified this delay by developing a classification scheme for discrete stages of meiosis. Immunofluorescence localization of RAD-51 protein revealed that delayed meiotic cells also contained persistent recombination intermediates. Through genetic analysis, we found that this cytological delay in meiotic progression requires double-strand breaks and the function of the crossover-promoting heteroduplex HIM-14 (Msh4) and MSH-5. Failure of X chromosome synapsis also resulted in impaired crossover control on autosomes, which may result from greater numbers and persistence of recombination intermediates in the delayed nuclei. We conclude that maturation of recombination events on chromosomes promotes meiotic progression, and is coupled to the regulation of crossover number and placement. Our results have broad implications for the interpretation of meiotic mutants, as we have shown that asynapsis of a single chromosome pair can exert global effects on meiotic progression and recombination frequency.

## Synopsis

Meiosis is a specialized cell division and an essential component of sexual reproduction. During meiotic prophase, each chromosome must pair with its unique homologous partner and undergo crossing over (genetic exchange) to segregate properly. A major mystery is how the molecular events of meiotic recombination are coupled to the large-scale dynamics of chromosome synapsis. This work reveals a link between the large-scale regulation of chromosome organization and the distribution of crossover events on the chromosomes. In *C. elegans,* defects in chromosome pairing or synapsis result in an extension of a normally transient stage of meiotic prophase. This study finds that this extension is associated with dysregulation of crossovers, so that more than the usual number of crossovers occur, and their distribution is shifted along the chromosomes. These observations contribute to our understanding of crossover control, which normally ensures accurate transmission of genetic information from parent to progeny.

## Introduction

Meiosis ensures the reductional division of a diploid genome into haploid complements. Proper meiotic segregation depends on pairing, synapsis, and crossing over between homologous chromosomes. In addition to promoting genetic diversity [1,2], crossing over enables the bi-orientation of homologs at metaphase I by establishing physical connections (chiasmata) between chromosomes [3]. Crossover recombination is therefore essential for proper meiotic chromosome disjunction.

The very low frequency of achiasmate chromosomes at metaphase I implies that a specific mechanism ensures the placement of at least one crossover per chromosome, called the “obligate crossover.” In principle, a minimum crossover number of one could be achieved with an unregulated, random process, if the number of crossovers was sufficiently high. However, most organisms have far too few crossovers for a Poisson process to ensure that each chromosome receives at least one [4].

In addition to this “obligate crossover” phenomenon, exchanges are also subject to genetic interference. This term describes the observation that crossovers are spaced farther apart from each other than would be expected if they occurred independently. In *Caenorhabditis elegans,* interference is extremely potent, limiting the number of crossovers per chromosome to exactly one. It has been proposed that these two facets of crossover control, genetic interference and the obligate crossover, are mechanistically linked [5,6]. A high rate of crossover attempts could give rise to a low but nonzero number of crossovers on all chromosomes if interference prevented most crossover attempts from being realized.

The mechanisms governing crossover control are not well understood. In *Saccharomyces cerevisiae,* recombination events destined to give rise to crossovers can be identified very early in meiotic prophase [7], but it is not known whether these events display interference when they first appear. The chromosomal localization of synapsis initiation complexes containing Zip2 and Zip3 shows a pattern consistent with interference, even in the absence of synapsis or crossover formation [8], supporting an early imposition of interference. Recent work has suggested that intact meiotic chromosome axes are necessary to mediate the high level of interference seen in C. elegans [9–11], but we still know very little about the mechanism by which this control is mediated.

Recent work in our laboratory has characterized the effects of two different mutations that specifically prevent synapsis of the X chromosomes, and thereby severely inhibit the formation of crossovers on one chromosome pair. Asynapsis can be caused by deletion of the X chromosome Pairing Centers, *cis*-acting sites that are required to stabilize homolog pairing and initiate synapsis [12]. Mutations in *him-8,* which encodes a protein that specifically binds to the X chromosome Pairing Center, have similar consequences for X chromosome synapsis and crossing over [13]. These mutations thus present an unusual opportunity to analyze the effects of asynapsis in the presence of fully functional SC components and recombination machinery.

Here we have analyzed the consequences of these mutations on the global progression of chromosome events during meiotic prophase. We demonstrate that asynapsis of X chromosomes results in a delay in the chromosome reorganization that is normally observed during meiotic prophase, and also disrupts the normal pattern of crossovers on the synapsis-proficient autosomes. These two overtly different consequences are linked by our observations that the delay in chromosome reorganization requires several molecular components involved in establishing crossover intermediates, indicating that unresolved intermediates may act to induce the delay. These results also suggest that the delay mitigates genetic interference, perhaps by extending the temporal window during which crossover attempts are actively made.

## Results

### Defects in X Chromosome Synapsis Result in a Global Delay in Nuclear Reorganization during Meiotic Prophase

To estimate the length of time each nucleus spends in a given meiotic substage, whole gonads of age-matched animals (20 h post-L4) were stained with DAPI and imaged by wide-field optical sectioning microscopy and image deconvolution. The C. elegans gonad contains nuclei at all stages of meiotic prophase arranged in a spatiotemporal gradient. Each gonad contained between 400 and 1,000 nuclei, with an average of 635 nuclei per gonad. Every nucleus in each imaged gonad was counted and classified into one of four meiotic prophase substages based on criteria illustrated in Figure 1. Previous work has documented the appearance of nuclei in the premeiotic germline and the “transition zone,” which corresponds to the stages of leptotene and zygotene, where pairing and synapsis are initiated. Transition zone nuclei have a distinct polarized appearance, with the chromosomes and the large nucleolus each displaced toward one side [14]. We further divided the pachytene stage into discrete early and late pachytene substages. Early pachytene nuclei retain a polarized appearance, but most of the chromosomes are clearly separated and synapsed. Late pachytene onset is defined by the disappearance of the polarized configuration of chromosomes. Although there is a general progression from one substage to the next as distance from the distal end of the gonad arm increases, we note that the physical range of each substage overlaps with adjacent stages. To obtain an accurate measure of stage duration, we carried out a systematic classification of all nuclei by stage.

**Figure 1:**
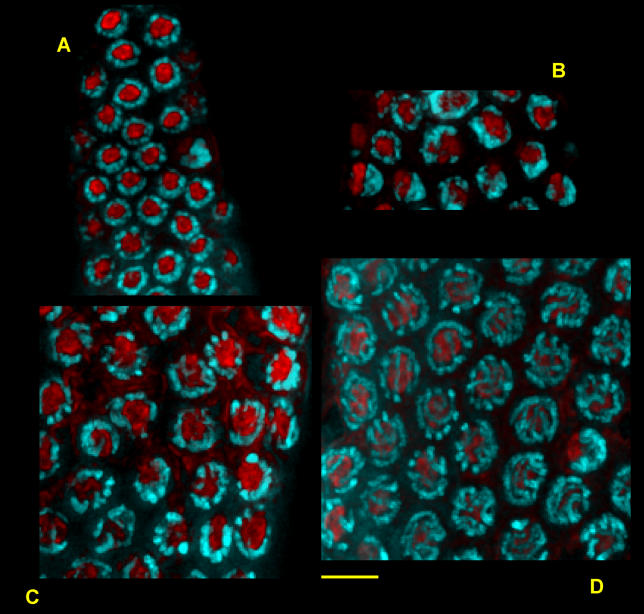
Criteria for Classifying Meiotic Nuclei into Substages of Prophase I (A) Nuclei with even distribution of chromatin that were located distal to transition zone nuclei were classified as premeiotic. (B) Nuclei with asymmetric distribution of chromatin that could not be resolved into individual chromosomes were classified as transition zone. (C) Nuclei with asymmetric distribution of individually resolvable chromosomes were classified as early pachytene. (D) Nuclei with fully paired chromosomes distributed around the entire nucleus were classified as late pachytene. DNA (blue) was stained with DAPI; nucleoli (red) were stained with antibodies against nucleolar protein NOP-1.

The fraction of nuclei that were classified as premeiotic and transition zone was not altered by mutations that perturb meiotic synapsis or crossing over, including *spo-11(ok79), msh-5(me23), him-8(mn253), rad-51(lg8701),* and *meDf2* (unpublished data). However, *him-8* and *meDf2* caused a clear shift in the relative numbers of early and late pachytene nuclei (Figure 2), suggesting that this particular transition is subject to regulation. As shown in Figure 2, *him-8* animals possess a significantly larger proportion of early pachytene nuclei, and a correspondingly lower fraction of late pachytene nuclei, relative to wild-type animals, indicating that the exit from the early pachytene stage is delayed in these mutants. *meDf2* gonads had an appearance qualitatively similar to that of *him-8* gonads (unpublished data). Chromosome synapsis occurs normally between autosomes in early pachytene in *him-8* (Figure 2, inset) and *meDf2* hermaphrodites, while the X chromosomes are unsynapsed. Therefore, a single pair of unsynapsed X chromosomes is sufficient to delay the early pachytene–late pachytene transition.

**Figure 2:**
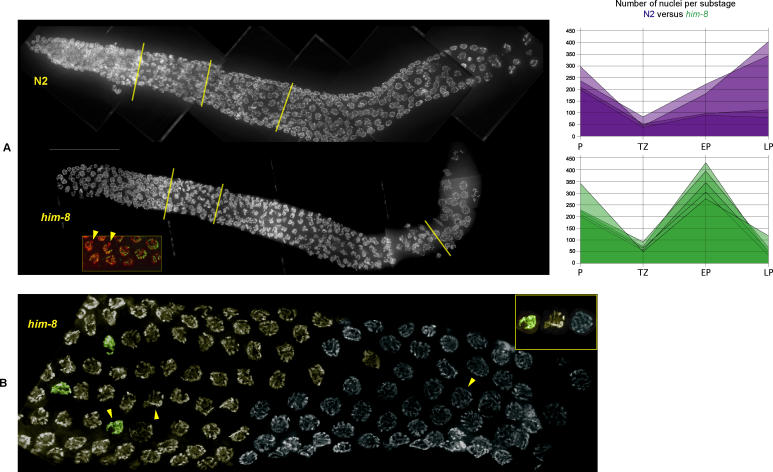
Progression from Early to Late Pachytene Is Delayed in X Asynapsis Mutants (A) Gonads imaged at 100× and composited. Top: N2; bottom: *him-8(mn253)*. The three yellow lines demarcate a rough separation of the gonad into four sections from left to right: premeiotic, transition zone, early pachytene, and late pachytene. Although each zone contains mainly nuclei of one meiotic substage, the transitions are not completely abrupt, necessitating counting of all nuclei in the gonad to obtain accurate staging. The *him-8* gonad contains a higher proportion of early pachytene nuclei, and a lower proportion of late pachytene nuclei, than the N2 gonad. Inset: synapsis is complete between autosomes in the early pachytene region in *him-8* gonads, whereas X chromosomes do not synapse (chromosomes, stained with DAPI, are displayed in red; the central element protein SYP-1, detected with immunofluorescence, is displayed in green; arrowheads mark the pycnotic X chromosomes which do exhibit SYP-1 staining.) Right: graph displaying raw numbers of nuclei at each substage for N2 and *him-8* gonads. Total numbers of nuclei are displayed for four (N2) or five *(him-8)* gonads. P, premeiotic; TZ, transition zone; EP, early pachytene, LP, late pachytene. (B) High-magnification view of transitions between meiotic prophase substages. Nuclei are tinted to highlight the classification of stage: green, transition zone nuclei; orange, early pachytene; blue, late pachytene. Arrowheads indicate exemplars of each stage, also shown from left to right in the inset (upper right). Scale bar, 50 μm.

### Persistence of RAD-51 Foci Corresponds to the Delay in Chromosome Reorganization

We used immunofluorescence to determine whether the increase in early pachytene duration is accompanied by perdurance of the recombination protein RAD-51 [15] on the chromosomes. Figure 3 displays a wild-type gonad (top), in which RAD-51 foci first appear in the transition zone, peak in early pachytene, and then largely disappear upon entry into late pachytene. In contrast, gonads from *him-8, msh-5,* and *him-8 msh-5* hermaphrodites (Figure 3, middle and bottom) contain RAD-51 foci throughout the delayed early pachytene stage as well as roughly halfway through late pachytene. We performed quantitation of RAD-51 localization in wild-type and mutants (Figure 3) and found that RAD-51 foci achieve higher absolute numbers, and persist through later meiotic substages, when all three mutant conditions were compared to wild-type. Significantly, in nuclei from the extended early pachytene region of *him-8* mutants, RAD-51 foci remain on the autosomes as well as the X chromosome (Figure 4). This demonstrates that recombination intermediates are more prevalent at later stages on autosomes, despite the availability of a synapsed homolog that should provide a template for recombinational repair. This observation suggested that the failed synapsis of the X chromosomes might influence the dynamics of recombination on the autosomes.

**Figure 3:**
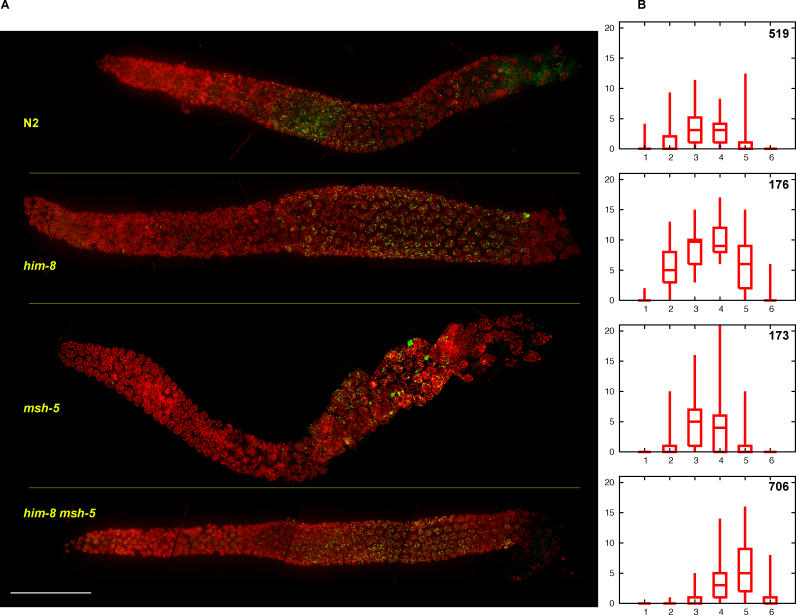
Progression of RAD-51 Focus Formation and Removal in Wild-Type and Meiotic Mutants (A) From top to bottom, gonads from wild-type (N2), *him-8, msh-5,* and *him-8 msh-5* worms are shown. In wild-type (top), RAD-51 foci appear in the transition zone and disappear in early pachytene. In all mutant conditions, RAD-51 focus formation begins in the transition zone, but persists throughout early pachytene, only disappearing at the very end of the gonad in late pachytene. Scale bar, 50 μm. (B) Quantitation of RAD-51 focus formation in wild-type and mutant conditions. Gonads were automatically divided into six equally sized regions, and nuclei assigned to each region based on their location. Graphs display box-whisker plots of focus numbers. The *x* axis indicates bins of equal length along the gonad; the *y* axis indicates the number of RAD-51 foci observed in a nucleus. The center horizontal line of each box indicates the median value; the box top and bottom indicate the first and third quartile values; the lines above and below the boxes extend to the entire range of measurements. Number of nuclei observed for each case are indicated at upper right.

**Figure 4:**
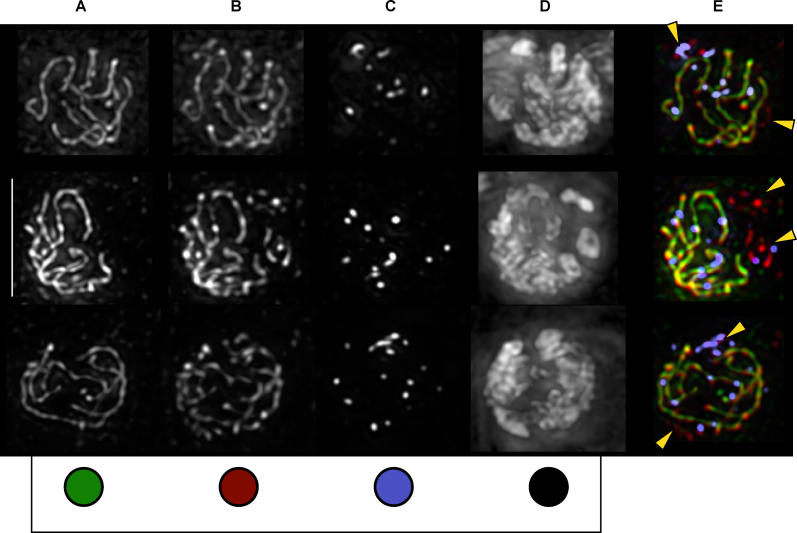
RAD-51 Foci Perdure on Synapsed Autosomes and Unsynapsed X Chromosomes in Extended Early Pachytene Three different nuclei from the extended early pachytene region of *him-8* gonads are shown, one on each row. Immunofluorescence of SYP-1, HTP-3, and RAD-51 is shown in columns A, B, and C; DAPI counterstaining of chromosomes is shown in column D; the colors of each component in the merged image in column E are indicated by colored circles below (green, SYP-1; red, HTP-3; blue, RAD-51; DAPI staining is not shown in the merged image). Chromosomes containing HTP-3 but not SYP-1 are the unsynapsed X chromosomes (arrowheads). RAD-51 foci are visible on both the X chromosomes and the autosomes. Scale bar, 5 μm.

This change in behavior of a recombination protein essential for meiosis [16] in C. elegans suggested a link between meiotic progression and recombination. To understand the relationship in further detail, we examined the effects of introducing mutations in other genes involved in recombination or DNA damage sensing.

### Delays in Meiotic Progression Require Components of the Crossover Machinery

To investigate the possible relationship between persistent recombination intermediates and the delay in chromosome reorganization we observed in *him-8* and *meDf2* mutants, we tested whether the delay required components that are important for recognizing DNA damage or processing double-strand breaks. We assessed whether such mutations affected the delay by staging entire gonads and comparing the ratio of early pachytene to late pachytene nuclei. Data are shown in Figure 5.

**Figure 5:**
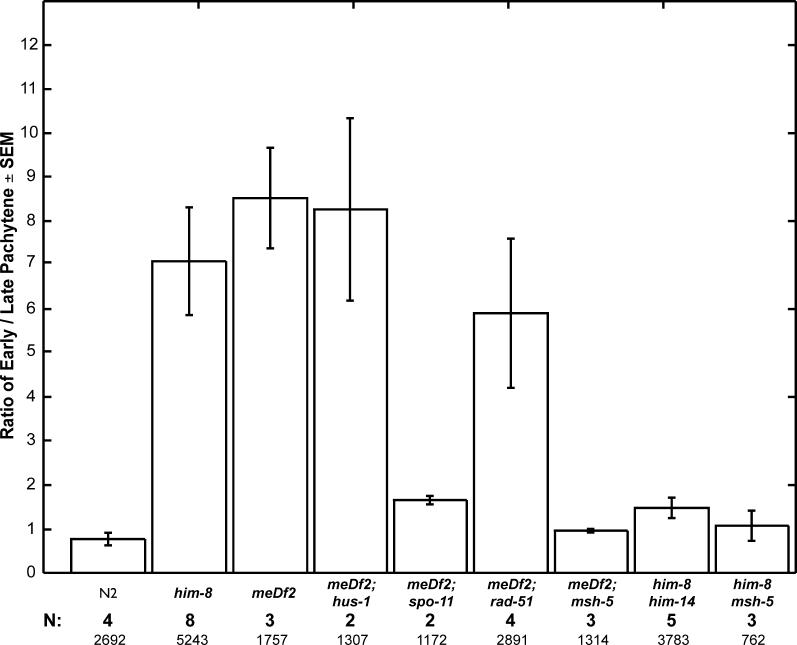
Ratios of Early Pachytene to Late Pachytene Nuclei in Wild-Type and Various Mutant Backgrounds Above each genotype analyzed, the mean early:late ratios are plotted; error bars indicate the standard error of the mean. The number of gonads scored (top row) and the total number of nuclei scored (bottom row) are indicated below the genotype.

It has been speculated that delays in reorganization of nuclear morphology might represent a response to unrepaired DNA damage, and we investigated this by crossing a *hus-1* mutation into *meDf2* and *him-8* mutants. Most mutations that disrupt the meiotic DNA damage checkpoint in C. elegans are also associated with defects in telomere maintenance and fecundity [17,18], but the *hus-1(op241)* point mutation, which we used, specifically abrogates its role in the meiotic DNA damage checkpoint. This *hus-1* mutation had no effect on the duration of polarized morphology in *meDf2* and *him-8* hermaphrodites, suggesting that the DNA damage checkpoint is dispensable for the delay in meiotic progression.

In contrast, mutations in genes required for the formation of double-strand breaks and crossovers did suppress the delay. Null alleles of *spo-11(ok79)* and *msh-5(me23),* as well as a temperature-sensitive allele of *him-14(it44)* (the C. elegans Msh4 ortholog) at the restrictive temperature all restored the wild-type ratio of early to late pachytene nuclei in the presence of unsynapsed X chromosomes. These results indicate that the presence of a molecular species that requires double-strand breaks and the Msh4/5 complex is required to maintain the early, polarized morphology of post-transition zone nuclei. Although HIM-14 and MSH-5 are required for crossover recombination, we currently do not have enough information to conclude that it is specifically the formation of crossovers, rather than another recombination intermediate involving the Msh4/5 complex, that removes the block to meiotic progression caused by asynaptic chromosomes. Further characterization of the Msh4/5 complex in the C. elegans germline is likely to shed light on this question.

Unlike mutations in *spo-11, him-14,* and *msh-5,* a null mutation in *rad-51(lg8701)* did not suppress the delay. This is surprising, as RAD-51 plays an early and essential role in double-strand break processing and is thought to mediate the assembly of recombination complexes, yet these results indicate that HIM-14/MSH4 and MSH-5 may play a RAD-51–independent role in mediating the delay. To test this possibility directly we used RNAi to inhibit *rad-51* expression in *him-8* and *him-8 msh-5* mutants. Like the *rad-51* mutation, RNAi-mediated knockdown of *rad-51* did not suppress the extended early pachytene zone in *him-8* mutants. In agreement with previous studies and our own analysis of *rad-51* mutants, reduction of *rad-51* function by RNAi did not in itself result in an extension of early pachytene. However, *msh-5(me23)* did suppress the delay in *him-8* mutants even when *rad-51* expression was inhibited through RNAi (unpublished data). Thus, we conclude that the HIM-14/MSH-5 complex is required to mediate the delay in early pachytene and that they must interact with the chromosomes in a *rad-51*–independent manner. This is consistent with previous observations that RNAi-mediated inhibition of *rad-51* had a different effect in *msh-5* hermaphrodites than in wild-type animals [16].

### Globally Altered Recombination Patterns on the Autosomes Result from Asynapsis of the X Chromosome

Our observation that asynapsis of the X chromosomes had a global effect on chromosome organization and on the abundance and timing of RAD-51 foci suggested that the autosomes might be affected by asynapsis of the X chromosomes, although their segregation is not markedly impaired [19]. Prior evidence has indicated that *him-8* can alter recombination in specific autosomal intervals. Specifically, crossover frequencies in specific intervals on Chromosomes I and III increase significantly in a *him-8(mn253)* mutant [20]. To test whether this effect is caused specifically by the *him-8* mutation, or results from chromosome asynapsis, we measured recombination in the *dpy-1* *unc-36* region on Chromosome III in both *him-8* and *meDf2*. In both *him-8* and *meDf2,* we observed an increase in genetic distance between *dpy-1* and *unc-36* similar to that observed previously. The distance increased from 17 centimorgans in wild-type (*n =* 1301) to 28 centimorgans (n *=* 1494) in *him-8,* and in *meDf2* the distance increased to 29 centimorgans (*n =* 963) (Figure 6A). We therefore conclude that the increase in genetic distance was not caused specifically by the *him-8* mutation, but by failure of the X chromosomes to synapse.

**Figure 6:**
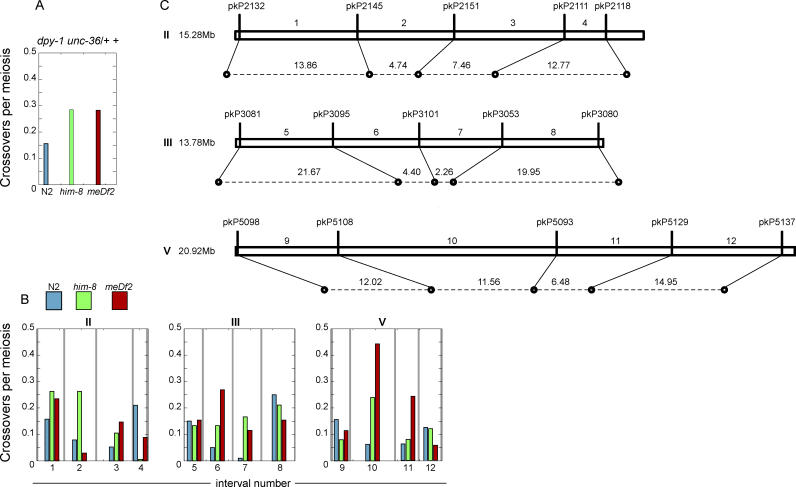
Crossover Alteration in X Chromosome Asynapsis Backgrounds Two genotypes *(him-8, meDf2)* were assayed for recombination by genetic crossing and SNP mapping. (A) The genetic distance between two visible markers on chromosome III was assayed by genetic crosses. Map distance increased from 17 centimorgans in N2, to 29 centimorgans in both *him-8* and *meDf2*. (B) Single-nucleotide polymorphism mapping of chromosomes II, III, and V. Five SNP markers were used, resulting in four intervals across the chromosome in which recombination could be assayed (*x* axis). The relative physical length of each region is shown by the distance between gray bars in the graph background. (C) Physical and genetic locations of single-nucleotide polymorphisms analyzed. The horizontal bars represent the physical length of the chromosomes (II, III, and V), with polymorphisms indicated above, proportional to their physical distance. Below each bar the polymorphisms are traced to a horizontal dashed line representing the interpolated genetic distance between them, also indicated numerically in centimorgans. Labels for each interval, numbered 1–12, correspond between B and C.

The increase in recombination between *dpy-1* and *unc-36* could reflect either a shift in the distribution of crossovers to favor the specific interval at the expense of other regions on Chromosome III. An alternative possibility is that autosomal recombination is globally increased in the presence of unsynapsed X chromosomes. To distinguish between these possibilities, and to examine the consequences of X asynapsis on other chromosomes, we measured the global number and distribution of crossovers on autosomes by single nucleotide polymorphism (SNP) mapping. Five primer pairs amplifying SNP-containing regions were chosen from the Washington University Genome Sequencing Center collection [21] to span the majority of each chromosome analyzed (Figure 6B). To generate worms for mapping experiments, *meDf2* and *him-8(mn253)* worms were repeatedly backcrossed to the Hawaiian strain CB4856 (Figure 6C) until all SNP markers converted to the Hawaiian alleles.

The genetic map lengths of Chromosomes II, III, and V increased in the X asynapsis mutants (Table 1), revealing a global increase in genetic exchange. Significantly, double crossovers were observed on Chromosome III in *him-8,* and on Chromosomes III, IV, and V in *meDf2* (Table 1), whereas no instances of double crossovers were detected for wild-type chromosomes, in accordance with many previous observations [9,10,22]. All mapping experiments that indicated double crossovers were verified by repeating the PCR and digestion on the same DNA sample. The presence of asynaptic chromosomes in a nucleus, therefore, impairs normal crossover interference on the other chromosomes.

**Table 1:**
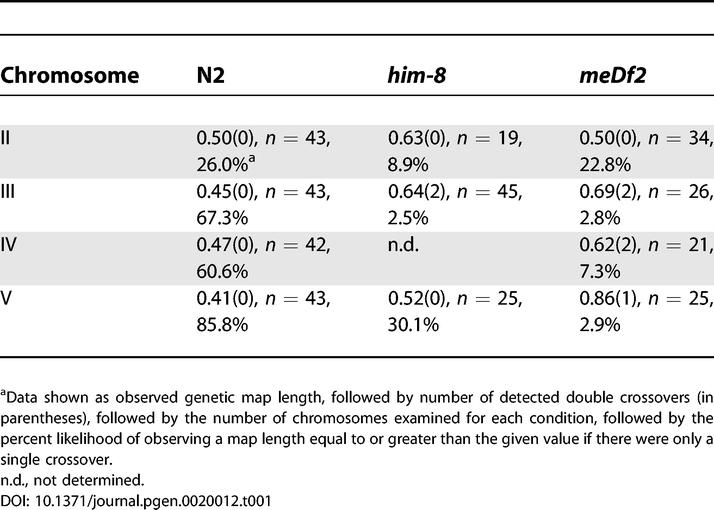
Genetic Recombination Alterations in X Asynapsis Mutants

The positional bias of crossover location was also altered in the X asynapsis mutants. Although all autosomes showed increased crossing-over when X chromosome synapsis was impaired, increases were not at all uniform over the intervals examined, and some intervals even showed reduced exchanges relative to wild-type. Chromosome II showed a pronounced polar shift in recombination toward the left side of the chromosome, which has been shown to contain the meiotic Pairing Center [23]. The left half of the chromosome showed an increased genetic map length of 52.6 MU, higher than the entire chromosome in wild-type hermaphrodites. In contrast, the combined map length of the two rightmost intervals dropped to only 10.0 MU, lower than the wild-type map distance of 20.23 MU (see Figure 6C). On Chromosomes III and V, the center intervals of the chromosome exhibited a significantly higher recombination frequency in X asynapsis mutants than in wild-type hermaphrodites. The result of this is to eliminate the normal bias toward higher recombination frequency near the chromosome ends seen in wild-type C. elegans meiosis. A similar disruption of this bias has been observed as a consequence of *rec-1* mutations in C. elegans [24]; the molecular basis of *rec-1* activity is still unknown.

## Discussion

We have shown that the failure of a single pair of chromosomes to synapse during meiotic prophase has far-reaching effects on the other chromosomes in the nucleus. The C. elegans Msh4/5 complex is involved in imposing a delay in the exit from early pachytene. The delay is associated with increased number and perdurance of RAD-51 foci, and increased autosomal recombination. While this study has used alterations of the X chromosome pairing center [25,13] to cause asynapsis of a single chromosome, earlier studies of other meiotic mutants such as *syp-1* and *syp-2* in C. elegans have reported an increase in the extent of the transition zone [14,26]. A simple explanation is that meiotic progression is delayed on a per-nucleus basis until key events such as recombination have been completed. The apparent action of this checkpoint between early and late pachytene suggests that this transition is a target for cell cycle regulatory machinery.

Based on observations that the number of RAD-51 foci in wild-type hermaphrodites peaks at early pachytene, and then steeply declines [15], and that RAD-51 foci persist longer when early pachytene is delayed (this study), we propose that there is a critical time period in early pachytene during which chromosomes can initiate and/or complete crossover recombination events, and that this period lapses upon entry into late pachytene. Therefore, nuclei that linger in early pachytene, i.e., nuclei containing unsynapsed chromosomes, have more opportunities for exchange to occur, through either initiation of new recombination events or conversion of existing recombination intermediates into crossovers. Our observation that this delay depends on the Msh4/5 complex strongly hints that the regulation of crossover recombination is linked to meiotic progression in wild-type meiosis. This hypothesis shares key features with a previous model proposing a “window of opportunity” during which double-strand breaks can be made on chromosomes [27]. Our results suggest that there may also be a “window of opportunity” for crossover formation, and that this window corresponds to the cytological appearance of polarized meiotic nuclei.

The mechanism by which the early, polarized pachytene configuration of chromosomes is converted into the late, dispersed configuration is not known. It has been proposed that recombinational events on chromosomes can affect their large-scale structure [28]. Alternatively, synapsis itself may be involved in driving the transition, as previously proposed. Further studies involving direct measurements of the physical properties of meiotic chromosomes may shed light on this question.

We have shown that meiotic progression delay in *him-8* and *meDf2* mutants requires HIM-14 and MSH-5, but not RAD-51. This implies that HIM-14 and MSH-5 (the C. elegans Msh4/5 complex) can act independently of RAD-51 to delay prophase progression, an unexpected result. It was previously shown that C. elegans MSH-5 can interact with meiotic chromosomes in the absence of RAD-51 [16]; we propose that in the absence of RAD-51, double-strand breaks are nevertheless processed into an intermediate that is bound by the Msh4/5 complex.

Previous work showed persistence of RAD-51 foci in the *msh-5* background [15]; we have shown here that foci also persist in *him-8* and *meDf2.* Loss of *msh-5* causes a return to wild-type meiotic progression, but also compromises RAD-51 focus removal. Since in no case do we observe RAD-51 focus removal from early pachytene nuclei before the transition to late pachytene, we conclude that the transition from early to late pachytene is necessary but not sufficient to enable removal of RAD-51 foci. If this is correct, then RAD-51 foci persist in *him-8* nuclei due to the failure to make the transition, and in *him-8 msh-5* nuclei due to the lack of MSH-5 protein.

Double crossovers were observed on autosomes in X asynapsis mutants, indicating that crossover interference is compromised. If the observed increase in autosomal recombination were brought about simply by an increase in the number of crossovers on chromosomes, we would expect with perfect sampling to observe double crossovers at a frequency equal to half of the increase in recombination. Our observations showed two out of 95 autosomes in *him-8,* and five out of 89 in *meDf2* with double crossovers, whereas none were detected for the 262 wild-type chromosomes we analyzed. Since previous studies of wild-type C. elegans meiosis have not detected double crossovers on autosomes, and only a very small number on the X chromosome [9,22], the modest level of autosomal double crossovers seen here shows a significant departure from the strict interference that is a hallmark of C. elegans meiosis.

The interchromosomal effect, wherein prevention of crossovers on one chromosome leads to alterations in crossing over on other chromosomes, was first described in *Drosophila melanogaster,* as a consequence of suppressed exchange on specific chromosomes due to structural heterozygosity [29–31]. A similar phenomenon called the intrachromosomal effect, wherein alterations to part of a chromosome increase the probability of crossover exchange on the unaltered part, has been documented in C. elegans [32], yeast [33], and *Drosophila* [34]. Our results show that global cytological differences in nuclear morphology and recombination protein dynamics correlate with conditions in which the interchromosomal effect is observed, supporting an earlier model [35] that the effect is due to a global delay in progression through meiosis, which lengthens the window of time during which recombination events can take place.

We have shown that the presence of unsynapsed chromosomes leads to both a delay in the normal progression of meiotic nuclear reorganization and to an increase in the amount of crossover recombination on the normally synapsed autosomes. The persistence of polarized chromosome morphology, and of RAD-51 foci on autosomes after they would normally have been cleared, suggests a global response of all the chromosomes triggered by asynapsis of a single chromosome pair. Two known meiotic checkpoints can be triggered by unsynapsed chromosomes in *C. elegans:* a DNA damage checkpoint [36] and the recently described asynapsis checkpoint [37]. However, the extension of the early pachytene region in *him-8* or *meDf2* mutants does not depend on *hus-1* (Figure 5), which is required for the DNA damage checkpoint; nor does it require the presence of an unsynapsed pairing center, which is required for the asynapsis-specific pathway. Thus, this delay in morphology may reflect a distinct meiotic checkpoint mechanism. The suppression of early pachytene extension by *him-14* and *msh-5* suggests that the delay is triggered by unresolved crossover intermediates whose formation is dependent on the Msh4/5 complex. In this model (Figure 7), the presence of unrepaired Msh4/5-containing intermediates triggers a checkpoint mechanism that acts to ensure the obligate crossover by delaying meiotic progression from early to late pachytene until a crossover has formed. This delay results in higher than normal accumulation of recombination intermediates, which may allow supernumerary crossovers to form on the synapsed chromosomes by overwhelming the normal operation of crossover interference. We conclude that this instance of the interchromosomal effect in C. elegans is best explained as an effect of the disruption of the normal timing of meiotic prophase events, and is not due to a direct, mechanistic effect of unsynapsed chromosomes on autosomes. Since recombinational failure has the potential to lead to aneuploidy and developmental defects, especially in humans (reviewed in [38]) it is critical to understand the feedback between molecular events on chromosomes and cell cycle progression in the germline. We have shown that one aspect of this feedback in C. elegans is linked to the process of crossover formation.

**Figure 7:**
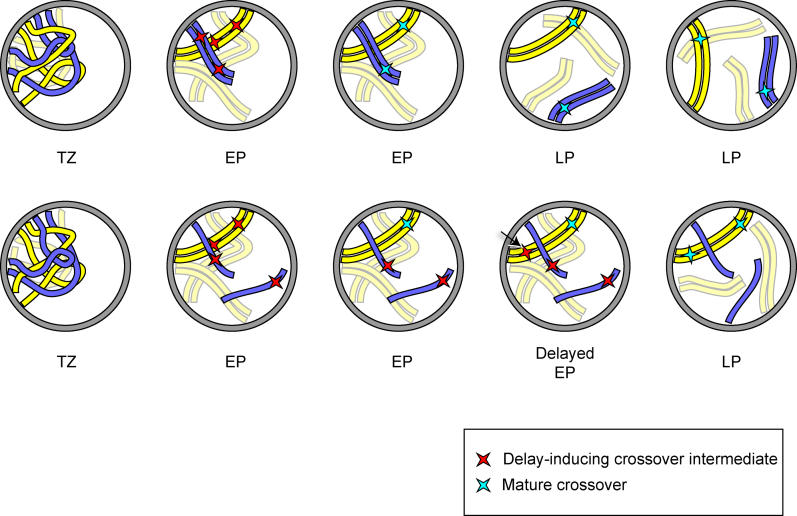
Model of Meiotic Progression in Wild-Type and X Asynapsis Mutants Top: in the wild-type situation, after initial pairing completes in the transition zone (TZ), multiple recombination events are initiated (red) on chromosomes as they adopt the early pachytene (EP) configuration. When all chromosomes have received a crossover (blue), recombination intermediates no longer inhibit forward progression in meiotic prophase, and chromosomes enter late pachytene (LP) and lose their polarized configuration. Bottom: in *him-8* and *meDf2* mutants, recombination events are initiated normally on both synapsed autosomes and the unsynapsed X chromosomes. The failure of recombination intermediates to resolve leads to a delay in the normal progression of meiosis, during which either additional recombination intermediates (arrow) can be initiated, or existing recombination intermediates can persist without being removed. Some proportion of these extra events may also lead to crossovers. Eventually, all recombination intermediates are cleared from both synapsed and unsynapsed chromosomes, and the polarized configuration is lost.

## Materials and Methods

### Genetic mapping.

Marker pairs *dpy-1(e1)* and *unc-36(e251),* in strain SP462, were crossed into *meDf2; mnDp66* and *him-8(mn253)* animals. These markers were then made heterozygous by crossing to *meDf2; mnDp66* or *him-8(mn253)* males. F1 hermaphrodite progeny of this cross were allowed to self and their progeny scored for *R,* the fraction of recombinant (Dpy non-Unc and Unc non-Dpy) progeny. Map distances *(p)* were calculated using the equation p=1−1−2R [39]. Genetic map lengths for SNP mapping experiments were calculated based on the fraction of recombinant chromosomes out of the total observed. Significance was assessed by estimating via simulation (program code available on request) the probability of observing a map length equal to or greater than a given value on each chromosome we analyzed, given the total chromosome length and the locations of SNP markers.

### Creating X asynapsis mutants in the Hawaiian background.

Male *him-8(mn253)* and *meDf2* worms were crossed to strain CB4856; cross progeny were picked to single plates and allowed to self. Ten hermaphrodites from the F2 generation were picked to single plates and scored for >30% male self-progeny. After five backcrosses, the strains were checked by single-worm PCR for the conversion of N2 to Hawaiian SNPs. The lack of uncut (N2) bands in all regions under consideration indicated backcrossing had gone to completion.

### DNA preparation and PCR.

To generate DNA for SNP analysis, single hermaphrodites heterozygous for Hawaiian and N2 markers were test-crossed to males of strain PD4792 homozygous for N2 SNPs. These males contained the insertion mIs11, enabling the identification of cross progeny by GFP fluorescence in the pharynx. Extensive testing showed no abnormal chromosome segregation in worms carrying one copy of mIs11 (unpublished data). Cross progeny were allowed to self 2–3 generations, at which point DNA was prepared. As a control, SNP analysis was also performed on single N2/CB4856 mosaic male worms. These worms were generated by crossing N2 males to single N2/CB4856 heterozygotes, which were generated by crossing CB4856 males to single N2 hermaphrodites. To prepare DNA, single males were frozen in 4 μL of lysis buffer (50 mM KCl, 10 mM Tris-HCl [pH 8.3], 2.5 mM MgCl_2_, 0.45% TERGITOL NP-40, 0.45% Tween 20, 60 μg/mL Proteinase K) at −80 °C for at least 1 h, then lysed at 60 °C for 1 h, followed by 15 min at 95 °C. Each lysate was mixed with 100 μL of a PCR mix (13 mM Tris-HCl [pH 8.3], 65 mM KCl, 3.2 mM MgCl2, 0.26 mM mixed dNTPs, 0.07 units Taq Polymerase), and 20 μL aliquots of this mix were transferred into five separate wells of a 96-well plate. Following this, the appropriate primers were separately added to each well (2.30 μL of a combined 4.4 μM forward primer, 4.4 μM reverse primer mix). The SNP-containing regions were amplified via touchdown PCR (10 cycles of a 67 °C to 62 °C touchdown: 94 °C 15s, 67 °C [−0.5/cycle] 30s, 72 °C 45s; 18 standard cycles: 94 °C 15s, 62 °C 25s, 72 °C 45s). Next, a digest solution (3.0 μL of 10× restriction enzyme buffer [New England Biolabs, Ipswich, Massachusetts, United States], 0.3 μL of 10 mg/mL BSA [if needed by enzyme], five units of restriction enzyme, and water to 10 μL) was added directly to the PCR solutions, which were then incubated at the appropriate temperature for at least 2 h.

### Imaging.

Gonads were dissected from worms in 1× egg buffer (25 mM HEPES [pH 7.4], 118 mM NaCl, 48 mM KCl, 2 mM MgCl_2_, 2mM CaCl_2_). For antibody staining, gonads on slides were fixed in 1% formaldehyde for 5–10 min, frozen on dry ice, and transferred to ethanol at −20. Slides were washed 3× for 10′ in 1× PBST (PBS with 0.1% Tween-20), blocked with 10 mg/ml BSA in PBST for 1–2 h, then incubated with an antibody against mouse α-NOP-1 overnight at room temperature. Slides were washed 3× in 1× PBST, then secondary antibody (Cy5-labeled goat α-mouse; Invitrogen, Carlsbad, California, United States) was applied in PBST for 2 h at room temperature. Three more washes in PBST were carried out, with the second wash containing 10 μg/ml 4′-6-diamidino-2-phenylindole (DAPI) to stain DNA. Slides were mounted in glycerol with 1% *n*-propyl gallate as an antifade agent. Slides were imaged with a Deltavision microscope system (Applied Precision, Issaquah, Washington, United States) using a 100×, 1.4NA lens (Olympus, Tokyo, Japan). Images were deconvolved with the conservative algorithm from the Priism suite [40]. Nuclei were scored by the appearance of chromosomes and the position of the nucleolus relative to the nuclear envelope, and labeled using the PickPoints program in the Priism software suite. Custom image processing programs, written in C (source code available upon request), were used to composite separate three-dimensional stacks into TIFF mosaic projections.

### RNAi inhibition of *rad-51* expression.

To inhibit *rad-51* expression by RNA interference, hermaphrodites were injected with 0.5 μg/ml double-stranded RNA complementary to the *rad-51* coding sequence. The template for in vitro transcription of this RNA was the *rad-51* clone from the Ahringer Lab RNAi library [41]. Heterozygous *him-8*/mIs11 or *him-8 msh-5*/mIs11 animals were injected and these P0s transferred to individual plates. Homozygous *him-8* or *him-8* *msh-5* progeny were selected based on the absence of *myo-2*::GFP expression in worms whose *myo-2*::GFP+ siblings produced only inviable eggs, indicating effective inhibition of *rad-51* expression. These *him-8*/+, *him-8* *msh-5*/+ or +/+ siblings were also dissected, fixed, and stained with DAPI, and showed univalent chromosomes and the presence of chromosome fragments at diakinesis, identical to the defects reported in both *rad-51*(lg8701) and *rad-51*(RNAi) hermaphrodites.

## Abbreviations

SNP: single nucleotide polymorphism
